# Machine Learning Models and Mathematical Approaches for Predictive IoT Smart Parking

**DOI:** 10.3390/s25072065

**Published:** 2025-03-26

**Authors:** Vesna Knights, Olivera Petrovska, Jasmina Bunevska-Talevska, Marija Prchkovska

**Affiliations:** 1 Faculty of Technology and Technical Science, University “St. Kliment Ohridski”—Bitola, 7000 Bitola, North Macedonia; 2 Faculty of Technical Sciences, Mother Teresa University, 1000 Skopje, North Macedonia; olivera.petrovska@unt.edu.mk; 3 Faculty of Technical Science, University “St. Kliment Ohridski”—Bitola, 7000 Bitola, North Macedonia; jasmina.bunevska@uklo.edu.mk; 4 Faculty of Computer Sciences, Mother Teresa University, 1000 Skopje, North Macedonia; marija.prckovska@students.unt.edu.mk

**Keywords:** machine learning, artificial intelligence, IoT integration, data analysis, predictive analytics

## Abstract

This paper aims to create an innovative approach to improving IoT-based smart parking systems by integrating machine learning (ML) and Artificial Intelligence (AI) with mathematical approaches in order to increase the accuracy of the parking availability predictions. Three regression-based ML models, random forest, gradient boosting, and LightGBM, were developed and their predictive capability was compared using data collected from three parking locations in Skopje, North Macedonia from 2019 to 2021. The main novelty of this study is based on the use of autoregressive modeling strategies with lagged features and Z-score normalization to improve the accuracy of regression-based time series forecasts. Bayesian optimization was chosen for its ability to efficiently explore the hyperparameter space while minimizing RMSE. The lagged features were able to capture the temporal dependencies more effectively than the other models, resulting in lower RMSE values. The LightGBM model with lagged data produced an R^2^ of 0.9742 and an RMSE of 0.1580, making it the best model for time series prediction. Furthermore, an IoT-based system architecture was also developed and deployed which included real-time data collection from sensors placed at the entry and exit of the parking lots and from individual slots. The integration of ML, AI, and IoT technologies improves the efficiency of the parking management system, reduces traffic congestion and, most importantly, offers a scalable approach to the development of urban mobility solutions.

## 1. Introduction

Cities and urban areas have many parking problems, including limited parking, the inefficient use of space, congestion, and more carbon emissions from vehicles searching for parking spaces. Studies have revealed that between 20 and 30% of urban traffic jams are a result of drivers in searching parking spaces [1,2,3]. Conventional parking management systems employ static signage, manual enforcement, and first-come-first-serve parking space management, which are ineffective in managing real-time parking demands. These inefficiencies not only result in economic losses but also lead to environmental pollution and driver frustration.

This study aims to explore the application of machine learning (ML) and Artificial Intelligence (AI) in the context of IoT-based smart parking systems to enhance the accuracy of parking slot predictions and thereby help reduce congestion [4,5]. This paper proposes the use of ML models that are trained in using real-time data from IoT sensors deployed within parking spots to predict occupancy trends and therefore provide drivers with predictive information on available parking spaces [6,7,8].

Machine learning has emerged as a powerful tool for analyzing and predicting complex data patterns across diverse domains [9], including agriculture, factory [10], healthcare [11,12], and autonomous systems [13]. Its adaptability and ability to handle large datasets have made it an indispensable component in solving real-world problems [14,15,16].

Recent advances have further expanded the application of ML techniques. For instance, ML-based guidance and control systems now enable more efficient operations in dynamic environments [17,18,19]. Additionally, ML models have proven to be effective in enhancing the security and functionality of IoT systems [20,21,22]. These developments underscore the versatility of ML in various fields, including cyber security, by detecting and mitigating vulnerabilities [23].

The evolution of algorithms has revolutionized problem-solving strategies, delivering innovative solutions that are both accurate and efficient. Foundational theories, such as Vapnik’s statistical learning theory, emphasize the balance between model complexity and generalization capabilities [24]. Furthermore, regression analysis remains a cornerstone technique, with traditional methods being enhanced by ML approaches to offer greater flexibility and predictive power [25,26,27].

The main objective of this research is to develop and analyze ML models for smart parking systems, with an emphasis on their mathematical foundations and predictive performance in improving urban infrastructure. The study focuses on implementing regression-based ML techniques, including random forests, and gradient boosting machines, to predict parking occupancy more accurately. To enhance forecasting accuracy, we integrate autoregressive modeling with lagged features and Z-score normalization, which significantly improves time series prediction performance.

Decision tree-based ensemble learning methods were chosen for this study due to their robustness, interpretability, and computational efficiency [28,29], making them particularly well-suited for real-time IoT-based applications.

Decision trees, introduced by Quinlan, utilize recursive partitioning for straightforward yet effective classification and regression [30]. Random forests, as developed by Breiman, aggregate multiple decision trees to improve predictive accuracy and mitigate overfitting [31]. Similarly, Friedman’s gradient boosting approach refines predictions by optimizing residual errors from earlier models, leading to superior performance [32].

Unlike deep learning models [33], which require extensive computational resources and hyperparameter tuning, tree-based methods such as random forest, gradient boosting, and LightGBM provide fast training and inference times while effectively handling structured datasets. Additionally, their ability to capture nonlinear relationships and process missing data efficiently makes them highly suitable for smart parking prediction tasks.

In contrast, neural network-based models, such as LSTMs, have demonstrated superior performance in time series forecasting, larger datasets, and high-dimensional problems. Their application in real-time IoT-based parking systems presents several challenges, including a high computational cost and complex hyperparameter optimization. Given the structured nature of our dataset and the need for real-time predictions, ensemble learning techniques provide a practical and scalable solution for urban parking management.

Analyzing model performance is an important part of the ML process that aids in the identification of the most suitable techniques and parameters. Measures like RMSE and MAE are useful in determining the accuracy and reliability of the model. The efficacy of these metrics in evaluating model performance is still under discussion in ongoing studies [34,35,36].

This research aims to comprehensively analyze ML techniques for smart parking systems, focusing on their practical applications, mathematical foundations, and performance evaluations. It implements autoregressive modeling with lagged features and Z-score normalization to improve time series prediction accuracy. By systematically analyzing these approaches, and comparing multiple ML models, the study identifies their superiority in time series prediction and their respective strengths and limitations, providing insights and recommendations for their effective implementation in smart city systems. Furthermore, it designs and deploys an IoT-based architecture that integrates ML predictions with real-world parking operations.

As a future area of study, robotic control systems can be integrated into parking management, leveraging nonlinear dynamics and machine learning algorithms to automate parking processes, optimize vehicle movement, and enhance decision-making [37,38].

## 2. Materials and Methods

The materials and methods used in this study include data collection, preprocessing techniques, and machine learning methodologies for predicting parking space availability and optimizing parking operations. A dataset was collected from three parking locations, processed to ensure data quality, and analyzed using three machine learning models using standard metrics. Regression-based ML models, random forest, gradient boosting, and LightGBM, are developed and their predictive capability is compared using the data collected.

### 2.1. Data Collection

In this study, the data were collected from three parking places situated in Skopje, Republic of North Macedonia for the time period of 2019–2021. The data include the following:

The total number of vehicles in the parking area was calculated as the sum of the initial number of parking spaces, plus the number of vehicles that entered, minus the number of vehicles that exited.

**Figure 1:**
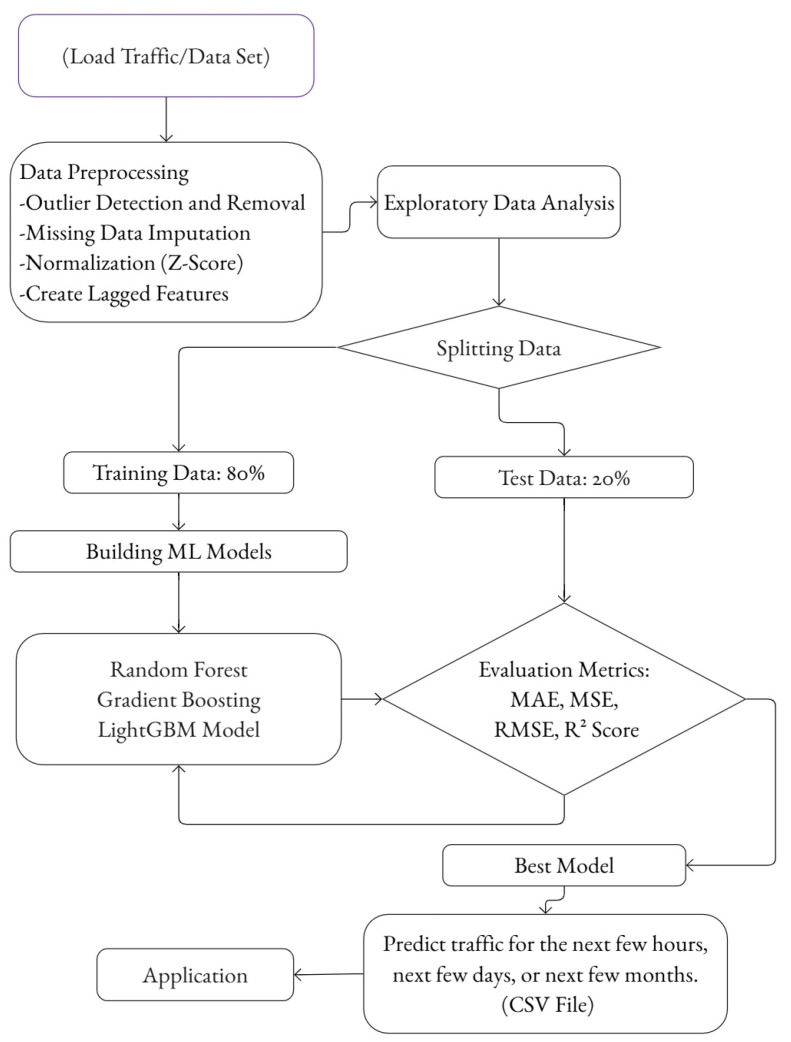
Machine learning methodology for traffic prediction in smart parking systems.

Figure 1 illustrates the workflow for traffic prediction using machine learning models, from data collection to model evaluation and deployment.

### 2.2. Data Preprocessing

To ensure data integrity and suitability for analysis, the following preprocessing steps were applied:

- Outlier Detection and Removal: Outliers were identified using *Z*-score analysis and handled to reduce their impact on the model’s performance.
- Missing Data Imputation: Values were imputed using forward-fill and backward-fill techniques.
- Normalization: *Z*-score normalization was used on the numerical features (such as vehicle counts and occupancy times) to make data more standard and thus improve model performance. Because these features were unbounded and of different scales, *Z*-score normalization allowed all the variables to contribute equally in the training process. Each feature value *x* was transformed using:

(1) $$z=\frac{x-mean(x)}{std(x)},$$

where x  represents the dataset, mean(x) is the average value of the feature, and $std\left(x\right)$ is the standard deviation.

- Lagged features were created to capture temporal dependencies in the data. For time series data, these were generated as follows:

(2) $$y_{t}=f(y_{t},\;y_{t-1},\;y_{t-2},\;\ldots \;y_{t-k})$$

where $y_{t}$ indicates that the value at time t is a function of its previous values up to k lags (k is the number of lagged values used as features). The function f can take various forms, such as a linear or nonlinear function.

If the summation form of the lagged features approach is applied, it leads to the autoregressive (AR) model (Equation (3)) when the relationship between current and past values is assumed to be linear. In this context, the AR model represents a specific mathematical realization of lagged feature usage, emphasizing the dependence.

(3) $$y_{t}=\sum_{i=1}^{k}\beta_{i}y_{t-1}+\varepsilon_{i}$$

Here, $\beta_{i}$ are the regression coefficients indicating the influence of past values, and $\varepsilon_{i}$ is the error term. The strength of dependency at each lag k is analyzed using autocorrelation:

(4) $$\rho_{k}=\frac{\sum_{t=k+1}^{n}(y_{t}-\bar{y})(y_{t-k}-\bar{y})}{\sum_{t=1}^{n}{\left(y_{t}-\bar{y}\right)}^{2}}$$

where $\rho_{k}$ is the autocorrelation coefficient at lag k,$\;\bar{y}$ is the mean of the time series, and n is the number of observations.

### 2.3. Machine Learning Models

To improve parking management operations and predict the availability of parking spaces, three machine learning models were built. The dataset is divided into training and test sets, with 80% of the data used for training and 20% used for testing. Three machine learning models, namely, gradient boosting, random forest and LightGBM, were developed using the training set to learn the patterns from historical traffic information. The models were evaluated on the test set by means of performance metrics such as mean absolute error (MAE), mean squared error (MSE), root mean squared error (RMSE), and the coefficient of determination (R^2^ score). These metrics were used to determine the accuracy and the ability to generalize each model. Some techniques used herein are established while others are homegrown, and for explanations, we will be using the following terms: N is the number of observations, y is the actual value (real observed value), $\hat{y}$ is the predicted value, and $\bar{y}$ is the mean (average) of actual values.

#### 2.3.1. Random Forest Regressor

The Random Forest Regressor is an ensemble learning algorithm based on decision trees. It is renowned for its ability to handle nonlinear relationships and complex datasets. The algorithm constructs multiple decision trees during training and combines their predictions through averaging or voting to improve accuracy. The random forest constructs multiple decision trees during training. The prediction of $\hat{y}$ for a given input x is obtained by averaging (or voting for) the predictions of individual trees [28]:

(5) $$\hat{y}=\frac{1}{N}\sum_{i=1}^{N}y_{i}$$

where *N* is the number of trees and $y_{i}$ is the prediction of the i-th tree.

#### 2.3.2. Gradient Boosting Model

This is machine learning built on concepts of partitioning and variance reduction (e.g., minimizing the sum of squared errors). Its formula primarily focuses on splitting criteria and predicting mean values within regions.

(6) $$F_{m}(x)=F_{m-1}(x)+{\gamma_{m}h}_{m}(x)$$

$F_{m}(x)$ is the updated model at iteration m, $F_{m-1}(x)$ is the previous model, $\gamma_{m}\;$ is the learning rate, and $h_{m}(x)$ is the weak learner (decision tree) fitted to the residuals.

Each iteration minimizes the residual errors:

(7) $$r_{i}=y_{i}-F_{m-1}(x_{i})$$

where ri is the residual at observation i.

A new tree hm(x) is fit to the residuals using the mean squared error (MSE) as the loss function:

(8) $$L(y,\hat{y})=\frac{1}{n}\sum_{i=1}^{n}{\left(y_{i}-{\hat{y}}_{i}\right)}^{2}$$

Each tree improves the overall prediction by correcting errors from previous trees. Split Selection is based on residual errors. Optimization is based on first-order gradient boosting.

#### 2.3.3. Light Gradient Boosting Machine (LightGBM) Regression Model

LightGBM (Light Gradient Boosting Machine) is a type of regression algorithm used for predicting continuous values. It is based on gradient boosting (Equation (7)).

LightGBM minimizes an advanced objective function with regularization:

(9) $$L(F)=\sum_{i=1}^{n}{\left(y_{i}-\hat{y}\right)}^{2}+\lambda \sum_{j}\omega_{j}^{2}$$

Using histogram-based feature selection for faster training, feature values *x* are grouped into bins indexed by *j*.

Instead of scanning every unique value, LightGBM finds the best bin j* to split using:

(10) $$\Delta Gain=\frac{1}{2}\left[\frac{G_{L}^{2}}{H_{L}+\lambda }+\frac{G_{R}^{2}}{H_{R}+\lambda }+\frac{{\left(G_{L}+G_{R}\right)}^{2}}{H_{L}+H_{R}+\lambda }\right]-\gamma$$

where $G_{L}\;$ and $G_{R}\;$ are the sums of gradients for the left and right child nodes after the split. $H_{L}\;$ and $H_{R}\;$ are the sums of Hessians for the left and right child nodes.

λ is the regularization parameter to prevent overfitting. γ is the pruning threshold (if the gain is too small, the split is discarded).

Split tree leaves with the highest gradient gain instead of level-wise growth. Optimize the objective function with second-order Taylor expansion

(11) $$L(y_{i},{\hat{y}}_{i})\approx L\left(y_{i},F_{m-1}(x_{i})\right)+g_{i}\left({\hat{y}}_{i}-F_{m-1}(x_{i})\right)+\frac{1}{2}h_{i}{\left({\hat{y}}_{i}-F_{m-1}(x_{i})\right)}^{2}$$

where $g_{j}=\frac{\partial L}{\partial {\hat{y}}_{i}}$ (first-order derivative), and $h_{j}=\frac{\partial^{2}L}{\partial {\hat{y}}_{i}^{2}}$ (second-order derivative, Hessian). The first term is the previous loss, the second term represents the gradient (first-order information), and the third term incorporates Hessian information, which measures how fast the gradient is changing.

#### 2.3.4. Hyperparameter Optimization

It is known that ensemble learning methods heavily rely on hyperparameter selection. To improve model performance, hyperparameter tuning was performed using Bayesian optimization. For the random forest, the parameters n_estimators, max_depth, and min_samples_split were optimized. Gradient boosting and LightGBM were tuned for learning_rate, max_depth, and the number of estimators. Bayesian optimization [39] was chosen for its ability to efficiently explore the hyperparameter space while minimizing RMSE.

### 2.4. Model Evaluation

The test set is used to evaluate the models with the help of some performance metrics like the mean absolute error (MAE), mean squared error (MSE), root mean squared error (RMSE), and coefficient of determination, also known as the R^2^ score.

- To find the mean squared error (MSE) [34,35], calculate the difference between each actual and predicted value, $e_{i}=\left|y_{i}-{\hat{y}}_{i}\right|$, then convert all errors to positive values and add up all absolute errors.
  (12) $$MSE=\frac{1}{N}\sum_{i=1}^{N}\left|\;y_{i}-{\hat{y}}_{i}\;\right|$$
- The root mean squared error (RMSE) [34,35] measures the average magnitude of the errors between predicted and actual values. It penalizes large errors more than MAE because it squares them before averaging. It is sensitive to outliers. It highlights large errors (which is useful for high-risk predictions).
  (13) $$RMSE=\sqrt{\frac{1}{N}{\sum_{i=1}^{N}\left(y_{i}-\hat{y}\right)}^{2}}$$
- The R^2^ Score measures how well the predictions match the actual values [34,35]. It compares the model’s errors to the errors of a simple mean-based model, and shows model fitting and explanatory power.
  (14) $$R^{2}=1-\frac{\sum_{i=1}^{N}{\left(y_{i}-\hat{y}\right)}^{2}}{\sum_{i=1}^{N}{\left(y_{i}-\bar{y}\right)}^{2}}$$

These metrics assess the accuracy and generalization capability of each model. The model with the best evaluation results is selected for deployment. The selected model generates traffic predictions for future time intervals, such as the next few hours, days, or months, and exports the results as a CSV file. The predictions are then used in a real-world application to support traffic management and decision-making processes.

### 2.5. Deployment and Integration

**Figure 2:**
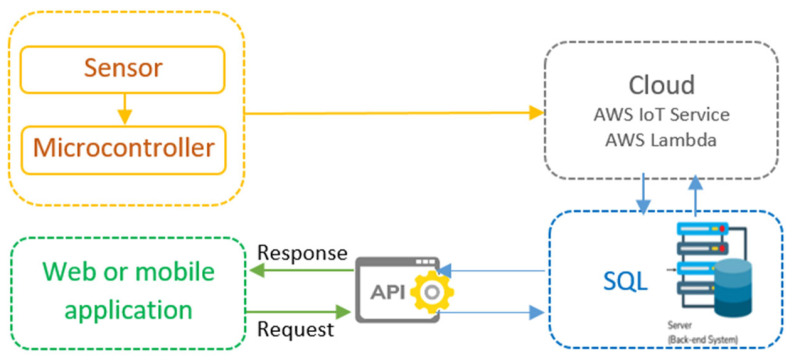
IoT data flow system for smart parking.

The real time prediction of parking availability is enabled by deploying the best performing model. Web APIs and web services are developed to enable third party access to prediction outputs. The integration of data analysis and machine learning with mobile applications, parking management systems, and digital signage makes the dissemination of the predictions to users seamless. The Internet of Things (IoT) is a system of interconnected devices that are able to collect, transmit, and process data in order to inform and enable better services and decisions. The IoT data flow has several components which include sensors, microcontrollers, cloud services, web applications, and databases (Figure 2). This architecture enables real-time management and data processing for smart parking operations. The sensor sends the parking data to the microcontroller which in turn sends the data to the AWS IoT Service through a cloud connection. The incoming data are received by AWS Lambda and saved in an SQL database for future use. Web or mobile applications communicate with the system through an API, which queries the cloud and receives responses with the information about the available parking spots.

**Figure 3:**
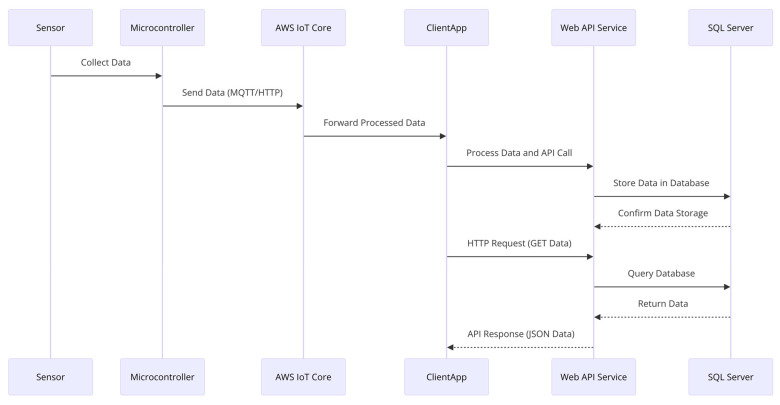
Sequence diagram of operational data flow in an IoT system for smart parking.

Figure 3 presents a sequence diagram illustrating the operational data flow in the IoT-based smart parking system, from sensor collection to database storage and client retrieval.

The process begins with the sensor collecting data and sending it to the microcontroller. The microcontroller sends the data to the AWS IoT Core through MQTT or HTTP and sends it to the cloud. The AWS IoT Core forwards the processed data to the ClientApp and an API call is made to the Web API Service (ASP.NET/Node.js) to store the processed data. The SQL Server receives the data and confirms its storage to the Web API. To retrieve data, the ClientApp makes an HTTP GET request to the Web API Service which queries the SQL Server and returns the data to the client in a JSON format.

## 3. Results

The results of the study include the evaluation of the three machine learning models, the analysis of their predictive performance, and the insights into the parking patterns derived from the dataset. The process begins with loading the traffic dataset, which is then subjected to a series of preprocessing steps. Outlier detection and removal, the imputation of missing values, normalization by Z-score, and the creation of lagged features to capture temporal dependencies are some of these steps.

### 3.1. Dataset

**Figure 4:**
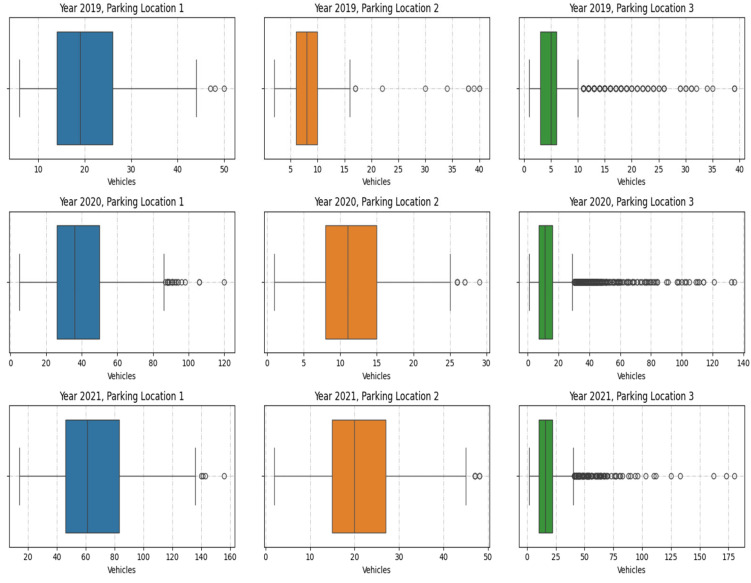
Boxplots of vehicle counts across parking locations and years.

The boxplots in Figure 4 below give a quick summary of the distribution of vehicle counts in three parking locations (1, 2, and 3) in the years 2019, 2020, and 2021. Each boxplot shows the central tendency, the variability, and potential outliers for vehicle counts during a particular year and at a particular location.

Parking Location 1: There is a greater extent of variability in vehicle counts in all three years. The median count increases from 2019 to 2021 which indicates that there is an increasing trend in parking demand. There are many outliers in 2020 and 2021, which suggests that there were some periods of very high parking activity now and then.

Parking Location 2: The boxplots shows a more consistent range of vehicle counts with less extreme values, suggesting that parking demand was stable over the years. The median does not change much, which means that there is not much temporal variation in the use of parking spaces.

Parking Location 3: The distribution is highly skewed, with a concentration of lower vehicle counts and numerous outliers. Outliers increase significantly in 2020 and 2021, which means that there were occasional spikes in vehicle counts that were not typical of the overall pattern.

#### 3.1.1. Normalization

**Figure 5:**
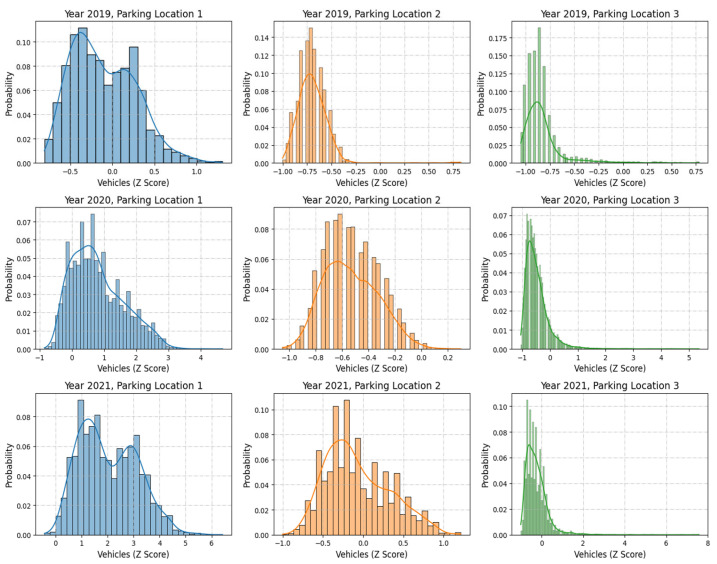
Z-Score-normalized distribution of vehicle counts across parking locations and years.

Figure 5 displays the z-score-normalized distributions of vehicle counts for the same parking locations and years as in Figure 2. Z-score normalization standardizes the data by centering it around 0 and scaling it to a standard deviation of 1, facilitating comparisons across locations and years.

- Parking Location 1 maintains a wider spread even after normalization, reflecting its inherently higher variability;
- Parking Location 2 shows a compact normalized range, emphasizing consistent parking patterns;
- Parking Location 3 remains skewed, with most values concentrated near the mean and a few extreme outliers.

Normalization aids in standardizing the data for machine learning models, ensuring equal weight for all features while retaining underlying patterns.

#### 3.1.2. Lagged Features

In this study, lagged values were generated to model the relationship between the number of vehicles at a specific time and preceding time intervals. The Autocorrelation Function (ACF) and the Partial Autocorrelation Function (PACF) are tools used to analyze time series data. They help us understand the relationship between a variable and its past values (lags) and are particularly useful for determining the structure of a time series model. The ACF measures the correlation between a time series and its lagged values. Significant spikes indicate strong correlations at those lags. The PACF measures the correlation between a time series and its lagged values after removing the influence of shorter lags [40,41]. Significant spikes indicate which lags have a strong direct relationship with the series.

Parking Place 1 shows significant autocorrelations for longer lags, indicating strong periodicity in parking patterns, suggesting a strong seasonal or cyclic pattern in vehicle counts. There are periodic fluctuations, indicating daily or weekly trends in parking occupancy.

In Parking Place 2, the ACF exhibits a sinusoidal pattern, indicating a repeating cyclic trend in vehicle counts. The oscillations suggest a strong periodicity, meaning that vehicle arrivals and departures follow a consistent time-based structure. The peaks and troughs indicate regular fluctuations in occupancy, possibly influenced by time-of-day or external factors like business hours.

In Parking Place 3, the ACF shows a clear wave-like pattern, indicating that the vehicle counts exhibit cyclic or seasonal behavior. Positive autocorrelation at shorter lags suggests that recent counts are positively related to previous counts.

The Partial Autocorrelation Function (PACF) plot (bottom panel) provides insights into the direct relationship between the current value and its lagged values while controlling for intermediate lags:

For Parking Place 1, the PACF plot shows strong correlations for the first few lags, emphasizing the influence of recent past values on current parking occupancy.

Parking Places 2 and 3 exhibit significant correlations for the initial lags, which diminish quickly, highlighting that only short-term dependencies are prominent for these locations.

The ACF and PACF analyses underscore the importance of incorporating lagged features into the machine learning models: Parking Places 2 and 3 benefit from models utilizing shorter lagged features due to their rapidly decaying correlations. Parking Place 1 demonstrates the need for models that are capable of capturing long-term dependencies to accurately predict future parking availability.

### 3.2. ML Models Results

Table 1 shows the performance metrics of three machine learning models used to make predictions of the availability of parking spaces in three different parking places, namely a Random Forest Regressor, a Gradient Boosting Model, and a LightGBM Regression Model.

The evaluation metrics are the coefficient of determination (R^2^) and the root mean squared error (RMSE). Furthermore, the models were tested in three ways: via their raw data, standardized using the Z-score, and their lag features.

- Random Forest Regressor: The best result was obtained using the lagged model for the first parking spot (R^2^ = 0.975874) and the use of Z-score normalization enhanced the accuracy of the third parking spot (R^2^ = 0.757917).
- Gradient Boosting Model: The lagged feature was found to perform best, with the highest R^2^ value of 0.967499 for the first parking spot and 0.883049 for the second parking spot. Z-score normalization was useful in reducing the RMSE.
- LightGBM Regression Model: The lagged feature model produced the best results in the predictive accuracy with an R^2^ value of 0.97725 for the first parking spot and 0.90499 for the second parking spot, with lower RMSE values.

**Figure 6:**
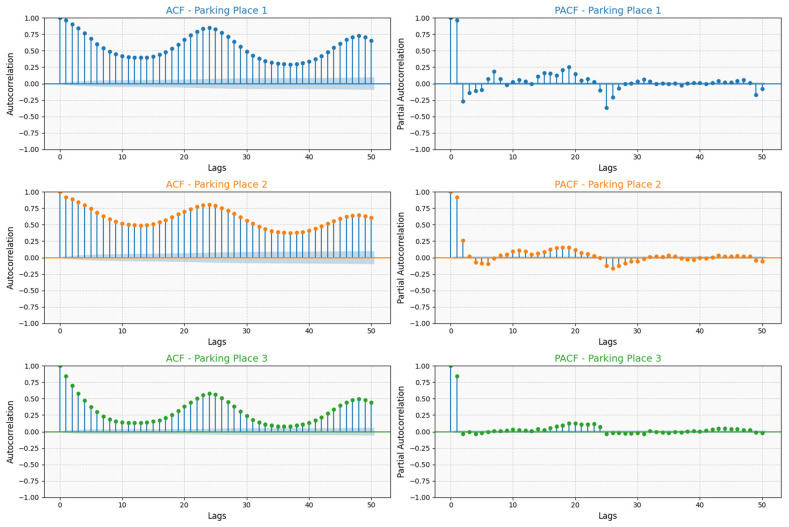
Autocorrelation and Partial Autocorrelation of vehicle counts across parking locations.

The ACF and PACF analyses (Figure 6) reveal valuable information about the time serial patterns in parking occupancy data. These insights are used to inform the effectiveness of lag-based models which include temporal effects. The predictive models are better able to capture temporal patterns by combining lagged features suggested by the ACF and the PACF with z-normalization. The performance of the random forest, gradient boosting, and LightGBM models with lagged features is consistent with the autocorrelation observed for each parking place.

Parking Place 1: Long-term correlations are evident from the ACF and PACF plots, hence lag-based models are most suitable for this dataset, especially with lagged features (LightGBM, R^2^ = 0.97725, RMSE = 0.15801) as random forest (R^2^ = 0.975874, RMSE = 3.970988) also learns from lagged features. Gradient boosting (R^2^ = 0.967499, RMSE = 4.092043) also uses lagged features but is not as efficient as LightGBM which suggests that LightGBM might be more efficient in capturing temporal patterns.

Parking Place 2: It has short-term cyclic patterns with high frequency. PACF plots show that lag-based models and the Gradient Boosting Model benefit most from lagged features (LightGBM, R^2^ = 0.90499, RMSE = 0.20100) and gradient boosting (R^2^ = 0.883049, RMSE = 2.484115).

Parking Place 3: The ACF and PACF plots reveal wave-like seasonal patterns with short-term correlations, which is in sync with the improvement in the lag-based models’ performance, while the efficiency of Z-score normalization shows that it is useful in dealing with seasonal effects.

### 3.3. Predictions

The models’ prediction accuracy is presented in Table 2, which shows the similarity in predictions for all the ML models (Random the Forest Regressor, the Gradient Boosting Model, and LightGBM Regression) with the use of lag features together with Z-score standardization. A best-performing model will be chosen for deployment to enable real-time parking availability predictions during the implementation of the algorithm. In this case, LightGBM Regression had the highest R^2^ Score and the smallest RMSE.

**Figure 7:**
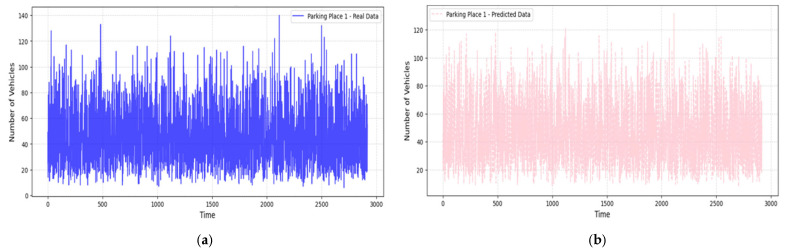
Real and predicted vehicle data for Parking Place 1: (**a**) real data collected over a period of 3000 h (four months); (**b**) vehicle data predicted using the LightGBM regression model.

**Figure 8:**
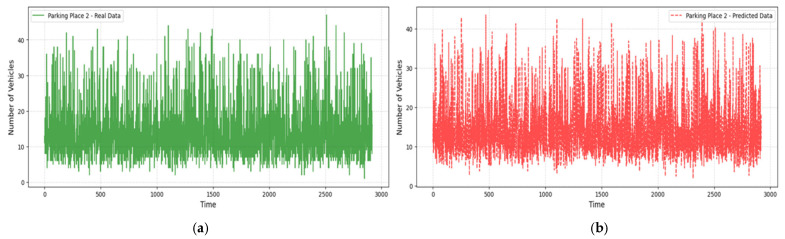
Real and predicted vehicle data for Parking Place 2: (**a**) real data collected over a period of 3000 h (four months); (**b**) vehicle data predicted using the LightGBM regression model.

**Figure 9:**
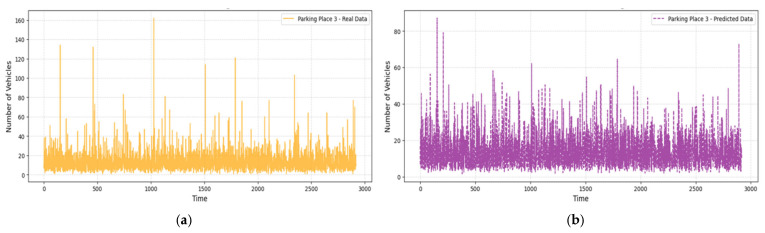
Real and predicted numbers of vehicle data for Parking Place 3: (**a**) real data collected over a 3000 h period; (**b**) predicted vehicle data obtained using the LightGBM regression model.

Figure 7, Figure 8 and Figure 9 compare real and predicted vehicle data for three parking places over four months. Figure 7 and Figure 8 also depict a high correspondence between the actual and predicted data, with the model capture the cycle of peak and off-peak hours of parking well. The R^2^ values were also high (0.905 for the Parking Place 2, 0.742 for the Parking Place 3, and 0.977 for the Parking Place 1) and the RMSE values were low (0.201, 0.392, and 0.158, respectively); thus, there is a good fit with minimal error. However, some differences can be seen in the third parking place (Figure 9), where RMSE is the highest. These discrepancies mean that model performance is different across locations and the most significant prediction errors are seen for the highest RMSE. Such deviations may be explained by more complex and less regular traffic flows in that area, which the model finds challenging to predict.

The LightGBM model demonstrates very strong generalization capability especially for Parking Places 1 and 2.

**Figure 10:**
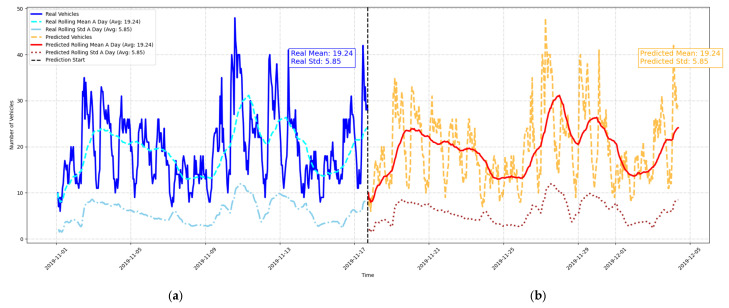
Real and predicted vehicle counts analysis: (**a**) real vehicle counts with their rolling mean and standard deviation for 15 days; (**b**) predicted vehicle counts with their corresponding rolling statistics for next 15 days.

The figure above (Figure 10) shows the client’s requested prediction of vehicle occupancy for the next 15 days based on the LightGBM model. The actual vehicle counts for the past 15 days are presented in the left panel, while the predicted occupancy for Parking Place 1 is presented in the right panel. The results show that the LightGBM model is capable of modeling both long-term trends, short-term fluctuations, and peak traffic events. It can be observed that the predicted rolling mean (red line) is fairly close to the actual rolling mean (cyan line) which indicates that the model is able to replicate the traffic patterns. Moreover, the predicted rolling standard deviation (brown line) follows the real standard deviation (light blue line) which means that the model is able to seize the variations and fluctuations.

The transition from training to forecasting is represented by the vertical dashed black line. The model has very good accuracy especially during the periods of low variability or stability. This performance is supported by the alignment of key statistical metrics: the predicted mean (19.24) and standard deviation (5.85) are close to the actual values, which indicates that the model is a good representation of the overall distribution of vehicle counts.

However, during the periods of rapid traffic changes, some deviations can be seen and these are indicated by the RMSE score. These discrepancies may suggest further possible enhancements, for instance, fine tuning the model with more temporal features or some other factors (e.g., weather or events).

### 3.4. System Design and Architecture

The system developed in this paper uses a Model-View-Controller (MVC) framework to manage data representation and database operations and user interaction. It also ensures that the structure is modular, scalable, and maintainable to incorporate machine learning predictions into the smart parking system.

**Figure 11:**
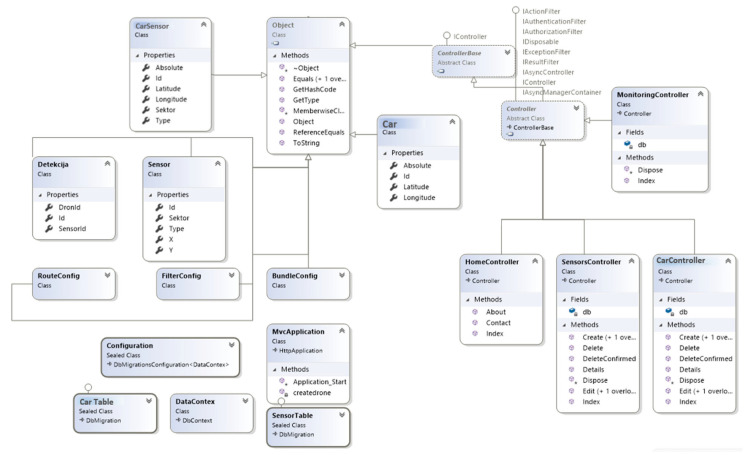
Class diagram of the smart parking management system implemented with the MVC framework.

Figure 11 shows the UML class diagram of the system and its elements and relationships in the context of the MVC architecture. The architecture is divided into three main classes of objects: entity classes (CarSensor, Car, Sensor, Detection) that identify the major objects and their states, where each class contains properties such as Latitude, Longitude, Type, Sektor, and SensorId; configuration and database classes (DataContex, CarTable, SensorTable) that support the data storage and transfer; and controller classes (HomeController, SensorsController, CarController) that handle the request, API call, and Create, Read, Update, and Delete operations.

The diagram is developed based on the MVC pattern, where controllers function as a go-between for the client’s requests and database operations; entity classes specify the type of data that is saved in the database; configuration is used to enable easy configuration and database migration.

**Figure 12:**
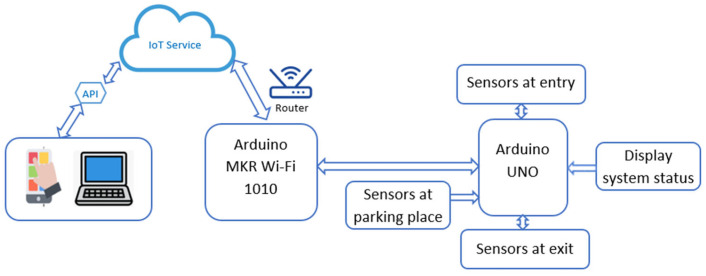
IoT-based hardware architecture for smart parking management.

This approach increases the scalability, maintainability, and modularity of the application. Figure 12 shows the hardware architecture of the smart parking system that employs IoT technologies for real-time data acquisition and analysis. The system uses sensors attached to an Arduino UNO to monitor the flow of vehicles at the entrance, exit, and within the parking area. Arduino MKR Wi-Fi 1010 sends the data to the cloud where it is further processed and made available remotely. Furthermore, a display system gives instant information to the users on the status of the system.

## 4. Discussion

The outcome of this research shows that regression machine learning models, specifically the LightGBM model, are suitable for the prediction of parking space availability in IoT-based smart parking systems. The results of the study show that the use of lag features and Z-score normalization [42] is appropriate for time series analysis in the modeling process. This approach is consistent with the principles of distributed lag models which are popular for incorporating temporal dynamics of explanatory variables in time series forecasting [43,44].

This analysis further shows that it is important to combine time series analysis tools (ACF and PACF) with machine learning models to capture both short-term patterns and long-term trends in the data to enhance the performance of smart parking prediction systems. According to Mills [45], autoregressive distributed lag (ARDL) models are very useful in distinguishing long-run relationships from short-run effects and this is in line with the concept of lag-based ML models such as LightGBM. In addition, the use of time series modeling and transfer function approaches has been found to be useful in many forecasting tasks [45,46]. The LightGBM model performed better than the other models in all the three parking spaces and had the highest R^2^ values and the lowest RMSE values. These results support the relevance of using autoregressive models in relation with ML frameworks for IoT-based traffic forecasting [45,46].

The result of this study shows that three machine learning models, i.e., the Random Forest Regressor, the Gradient Boosting Model, and the LightGBM Regression model, are efficient in predicting parking space availability based on time series data. Out of the three, the LightGBM Regression model had the best prediction accuracy with an R^2^ value of 0.97725, an RMSE of 0.158, and an MAE of 1.973. This is mainly because it has a high capacity to learn the temporal dynamics and makes robust forecasts with fewer prediction errors. The Random Forest Regressor model also performed reasonably well with the lag-based modeling (R^2^ = 0.97587, RMSE = 3.970); however, its higher MAE of 2.115 suggests that it is not as consistent as the other two models. The Gradient Boosting Model had an R^2^ value of 0.96749, an RMSE of 4.092, and an MAE of 2.278. The random forest, Gradient boosting, and LightGBM models were compared and it was seen that the inclusion of lag features improved the model performance [45].

From a mathematical perspective, ensemble learning integrates simple decision tree classifiers in a serial or parallel structure to approximate a complex nonlinear function. While it is true that deep learning models, particularly neural networks (NNs), can outperform tree-based models in certain scenarios, our approach used autoregression and Bayesian optimization to enhance predictive accuracy and efficiently capture temporal dependencies. Dujić Rodić et al. [47] reported that their Hidden Markov Model (HMM) and neural network (NN) achieved the best occupancy classification rate of 97% using LoRa sensors. Our LightGBM model provided very similar accuracy and had lower errors in prediction models.

Furthermore, while Dujić Rodić et al. aimed at classifying occupancy levels through signal intensity, we went beyond that to explore the use of regression models for time series forecasting in the context of smart parking systems and thus offer a more general-purpose solution for real-time control of parking spaces. 

Moreover, comparing our results with those reported by Kalbhor et al. [48] in their PARKTag system, which integrated AI and blockchain for parking management, our LightGBM model outperformed their machine learning classifiers in time series prediction, except for the SVM model used by Kalbhor et al., which achieved slightly better classification accuracy through blockchain integration. However, our study surpasses theirs in regression-based forecasting capabilities, which are essential for real-time occupancy prediction and resource allocation in smart parking management.

The deployment of the best-performing model within the IoT-based smart parking architecture demonstrates its practical applicability. The implementation of an IoT-based hardware architecture, consisting of Arduino MKR Wi-Fi 1010 and Arduino UNO, successfully enabled real-time data collection from entry, exit, and individual parking spaces. Similarly to the approach discussed by Rupani and Doshi [49,50,51], where IoT technologies such as sensors, actuators, and mobile applications facilitated automated parking slot reservations and reduced the time spent searching for parking, our system leverages real-time data transmission to enhance user convenience.

Additionally, the ASP.NET MVC-based software (version 5) architecture facilitated the real-time dissemination of predictions through web APIs, ensuring system modularity and scalability. This contrasts with the approach of Alharbi et al. [52], who focused on an OCR-based web application for parking management but lacked web API integration.

Despite the strong performance of the LightGBM model, slight deviations were observed, particularly in the Parking Place 3, where traffic patterns were more irregular. These discrepancies indicate that the model’s performance can be improved by incorporating additional external features, such as weather conditions, public event schedules, and real-time traffic conditions. Furthermore, as noted by Booth [53], forecasting models that incorporate structural modeling with exogenous variables, such as traffic patterns or weather conditions, can further enhance predictive accuracy, which is a potential area for future improvement in smart parking systems.

Future research directions could focus on investigating edge computing solutions to process predictions locally on IoT devices, thereby reducing latency and enhancing real-time responsiveness. As emphasized by Butt et al. [54,55], edge computing offers significant advantages in distributed processing. Additionally, expanding the study to incorporate robotic control systems for automated vehicle guidance within parking facilities could further enhance operational efficiency and automation [56].

## 5. Conclusions

The primary contribution of this paper is the enhancement of predictive accuracy by employing advanced regression models and time series analyses.

This paper highlights the possibility of applying machine learning approaches in conjunction with IoT applications for the effective management of parking spaces. The proposed methods not only improve the parking operations but also help in solving the problem of traffic jams and increase the effectiveness of smart city infrastructures.

From a practical point of view, the proposed system presents several benefits for the management of urban traffic flows, including the better use of resources, less congestion, and an improved user experience through real-time information of the availability of parking spaces. In addition, the modular structure of the system means that it can be expanded easily to encompass further car parks or linked with other third-party applications.

## Figures and Tables

**Table 1:** Features in the dataset.

Feature Name	Description	Type
**DateTime**	Timestamp of the recorded parking data	Datetime
**Hour of the Day**	Extracted hour from DateTime	Integer (0–23)
**Day of the Week**	Extracted day from DateTime (0 = Monday, 6 = Sunday)	Integer (0–6)
**Month**	Extracted month from DateTime (1 = January, 12 = December)	Integer (1–12)
**ParkingPlace**	Unique identifier for each parking location	Categorical (Integer)
**ID**	Unique record identifier (YYYYMMDDHH Parking Location)	String
**Vehicles**	Number of vehicles counted at that timestamp	Integer
**Entry Count** (Derived)	Vehicles entering the parking lot in that interval	Integer
**Exit Count** (Derived)	Vehicles leaving the parking lot in that interval	Integer
**Total Vehicles in Parking Lot** (Derived)	Current number of parked vehicles, calculated as Total Vehicles = Initial Capacity + Entry Count − Exit Count	Integer
**Occupancy Rate (%)** (Derived)	Parking space utilization percentage, calculated as	Float
	$\mathrm{Occupancy Rate}=\left(\frac{\mathrm{Total Vehicles}}{\mathrm{Total Parking Capacity}}\right)\times 100$	

**Table 2:** Performance metrics of machine learning models for smart parking prediction.

Parking Place No./Random Forest Regressor	R^2^	RMSE	MSE
1st	0.947253	5.292016	3.969012
2nd	0.865725	2.654923	1.991192
3rd	0.706305	5.318437	3.988827
**Random Forest Regressor—Z-Score Normalization**	**R^2^**	**RMSE**	**MSE**
1st	0.947843	0.263191	0.197393
2nd	0.869550	0.134316	0.100737
3rd	0.757917	0.243712	0.182784
**Random Forest Regressor—lag_model**	**R^2^**	**RMSE**	**MSE**
1st	0.975874	3.970988	2.978241
2nd	0.881247	2.522727	1.892045
3rd	0.749207	4.967311	3.725483
**Gradient Boosting Model**	**R^2^**	**RMSE**	**MSE**
1st	0.779331	8.877456	6.658092
2nd	0.727689	3.820714	2.865535
3rd	0.415435	8.173594	6.130195
**Gradient Boosting Model—Z-Score Normalization**	**R^2^**	**RMSE**	**MSE**
1st	0.94316	0.26166	0.196245
2nd	0.85673	0.13271	0.099532
3rd	0.69188	0.27928	0.209459
**Gradient Boosting Model—lag_model**	**R^2^**	**RMSE**	**MSE**
1st	0.967499	4.092043	3.069032
2nd	0.883049	2.484115	1.863086
3rd	0.718058	5.396979	4.047734
**LightGBM Regression Model**	**R^2^**	**RMSE**	**MSE**
1st	0.794175	3.300778	2.475583
2nd	0.571899	3.827113	2.870334
3rd	0.500927	2.587724	1.940793
**LightGBM Regression Model—Z-Score Normalization**	**R^2^**	**RMSE**	**MSE**
1st	0.947222	0.252000	0.189000
2nd	0.852718	0.134618	0.100963
3rd	0.704462	0.392441	0.294330
**LightGBM Regression Model—lag_model**	**R^2^**	**RMSE**	**MSE**
1st	0.977259	0.158018	0.118513
2nd	0.904999	0.201007	0.150755
3rd	0.741877	0.293423	0.205067

## Data Availability

The original contributions presented in the study are included in the article; further inquiries can be directed to the corresponding author.
